# Delayed CPK peak after primary PCI in STEMI: marker of reperfusion quality and short-term outcomes

**DOI:** 10.1186/s12872-025-05416-x

**Published:** 2025-12-12

**Authors:** Eitaro Umehara, Yutaro Nagase, Shunpei Yao, Kana Nagasawa, Noriaki Kobayashi, Yoshiki Asakura, Atsushi Miyajima, Naoto Inoue, Arata Hagikura, Takanori Kusuyama, Hidetaka Iida

**Affiliations:** Department of Cardiology, Tsukazaki Hospital, 68-1 Waku, Aboshi-ku, Himeji-city, Hyogo-prefecture 671-1227 Japan

**Keywords:** TIMI flow, Time to peak CPK, Primary PCI outcomes, Biomarker kinetics

## Abstract

Time to peak creatine phosphokinase (CPK) has historically been used as a surrogate marker of reperfusion in ST-elevation myocardial infarction (STEMI), but its significance in the contemporary primary percutaneous coronary intervention (PCI) era remains uncertain. We retrospectively analyzed 239 consecutive STEMI patients who underwent primary PCI at Tsukazaki Hospital between January 2021 and December 2024. Patients were categorized according to final post-PCI TIMI flow: TIMI 3 (Group A, n=194) and non-TIMI 3 (Group B, n=45). The median time to peak CPK was significantly shorter in Group A compared with Group B (365.0 vs. 684.0 minutes, p<0.001), whereas peak CPK levels did not differ significantly between the groups. At 30 days, the incidence of major adverse cardiovascular events plus congestive heart failure was significantly lower in Group A than in Group B (4.1% vs. 13.3%, log-rank p=0.01). These findings suggest that optimal post-PCI TIMI 3 flow is associated with faster enzymatic washout and better short-term outcomes, and that time to peak CPK may provide complementary information regarding reperfusion quality in STEMI patients.

## Introduction

Prompt and effective reperfusion is essential for improving clinical outcomes in patients with ST-elevation myocardial infarction (STEMI). Primary percutaneous coronary intervention (PCI) has become the standard therapy to restore coronary flow. However, despite successful angiographic results, truly effective reperfusion is not always achieved, because residual microvascular dysfunction and distal embolization can compromise myocardial salvage [1]. Biomarkers such as creatine phosphokinase (CPK) and cardiac troponins have traditionally been used to estimate infarct size. Among them, the timing of peak CPK has served as a surrogate marker for the reperfusion process. Earlier peaks are generally interpreted as reflecting more successful reperfusion and faster washout of necrotic enzymes, while delayed peaks may suggest impaired microvascular flow or ongoing myocardial injury [2].

Most studies addressing the clinical implications of time to CPK peak were conducted during the fibrinolytic era, and evidence in the contemporary PCI era remains limited [3]. In particular, procedural factors such as distal embolization and side branch occlusion can affect coronary flow and influence biomarker kinetics independently of infarct size [4–6].

The Thrombolysis in Myocardial Infarction (TIMI) flow grade is a widely accepted angiographic measure of coronary reperfusion, with TIMI 3 flow representing optimal restoration of epicardial flow [7]. Whether differences in post-PCI TIMI flow grades are associated with distinct patterns in CPK kinetics has not been thoroughly investigated in the current practice of primary PCI.

Therefore, in the present study, our primary objective was to evaluate the relationship between final post-PCI TIMI flow grade and the time to peak CPK in patients with STEMI. As a secondary and exploratory objective, we assessed whether delayed CPK peak timing was associated with short-term adverse clinical outcomes.

## Method

### Study population

**Fig. 1:**
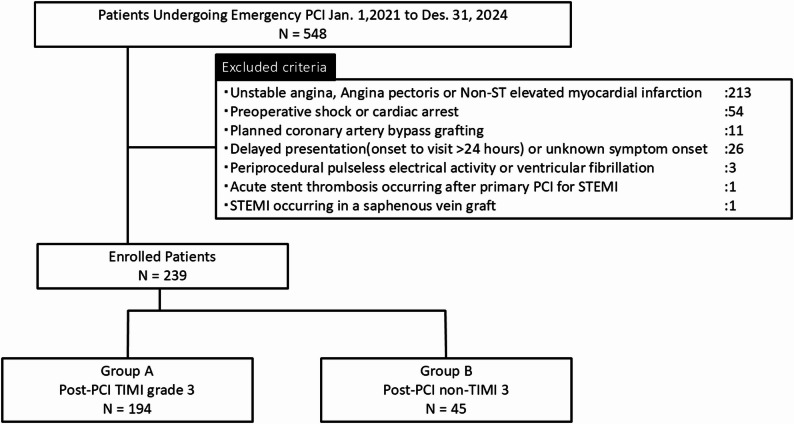
Study enrollment and exclusion. Flow chart showing the enrollment of STEMI patients undergoing primary PCI between January 1, 2021, and December 31, 2024. Of 548 patients initially screened, 309 were excluded according to predefined criteria, including unstable angina, non-ST elevation myocardial infarction, pre-procedural cardiac arrest, delayed presentation, and other conditions. The final study population included 239 patients, categorized into Group A (TIMI 3 flow, *n* = 194) and Group B (non-TIMI 3 flow, *n* = 45) according to final post-PCI TIMI flow grade

We conducted a retrospective, single-center study including patients with STEMI who underwent primary PCI between January 1, 2021, and December 31, 2024, at Tsukazaki Hospital. STEMI was diagnosed according to contemporary guideline-based criteria, including: ST-segment elevation ≥ 1 mm in ≥ 2 contiguous limb leads, and sex- and age-specific thresholds in leads V2–V3 (≥ 2.5 mm in men < 40 years, ≥ 2.0 mm in men ≥ 40 years, ≥ 1.5 mm in women), or new-onset left bundle branch block, together with symptoms consistent with myocardial ischemia and elevated serum troponin. Among 548 consecutive patients who underwent emergent PCI, 239 were included after applying the following exclusion criteria: unstable angina, angina pectoris, or non-STEMI (*n* = 213); pre-procedural cardiogenic shock or cardiac arrest (*n* = 54); planned coronary artery bypass grafting (*n* = 11); delayed presentation (> 24 h) or unknown symptom onset (*n* = 26); periprocedural pulseless electrical activity or ventricular fibrillation (*n* = 3); acute stent thrombosis after primary PCI for STEMI (*n* = 1); and STEMI in a saphenous vein graft (*n* = 1). Of the 239 patients included, post-PCI TIMI flow was dichotomized into two groups: TIMI 3 (Group A, *n* = 194) and non-TIMI 3 (Group B, *n* = 45) (Fig. 1).

### Data collection

Baseline demographic characteristics, cardiovascular risk factors, medical history, and pre-admission medication use were extracted from electronic medical records. Procedural information, including the culprit vessel, use of an intra-aortic balloon pump (IABP), and occurrence of distal embolism, was also collected. Laboratory data obtained at admission included CPK, CK-MB, troponin I, and B-type natriuretic peptide (BNP) or N-terminal pro-BNP (NT-pro BNP). For patients with BNP data only, NT-pro BNP values were estimated using the Ishihara conversion formula, which incorporates body mass index (BMI) and estimated glomerular filtration rate (eGFR) [8]. Pre-procedural and post-procedural TIMI flow grades were assessed by experienced interventional cardiologists blinded to patient outcomes and not involved in the index PCI procedure. Distal embolism was defined as any of the following: [1] development of slow-flow or no-reflow during PCI, excluding transient filter-related no-reflow associated with distal protection devices; [2] persistent slow-flow or no-reflow at the end of the procedure requiring intracoronary vasodilators (nicorandil or nitroprusside); [3] angiographically visible distal migration of thrombus (large thrombus embolization) that did not resolve despite appropriate mechanical or interventional measures, including thrombus aspiration, plain old balloon angioplasty (POBA), stenting, or dottering. This composite definition was selected to capture clinically meaningful embolic phenomena beyond vasodilator-requiring no-reflow alone. Side branch occlusion was defined as complete loss of flow in a side branch ≥ 1.5 mm in diameter on the final angiographic assessment. Serial CPK levels were measured every 3 h post-PCI until the peak was confirmed, followed by measurements every 12 h. Time to peak CPK was recorded as the interval (in minutes) from the end of the PCI procedure to the highest recorded CPK level. This sampling frequency reflects routine post-PCI practice at our institution and, although commonly used in several Japanese PCI centers, is not mandated as a national standard. The standardized protocol allowed precise determination of biomarker kinetics in this study. In addition to total CPK, CK-MB levels were measured in routine clinical practice and were available in 232 of the 239 patients. Because CK-MB sampling was not performed at identical time intervals to total CPK, CK-MB was evaluated as a complementary marker in a prespecified secondary analysis. Time to peak CK-MB was defined as the interval from the end of PCI to the highest measured CK-MB value.

### Clinical endpoints

The primary endpoint of this study was the time to peak CPK according to final post-PCI TIMI flow grade, reflecting enzymatic washout and reperfusion quality.

The secondary, exploratory clinical endpoint was the 30-day incidence of major adverse cardiovascular events (MACE) plus congestive heart failure (CHF).

MACE was defined as a composite of cardiac death, recurrent myocardial infarction, target vessel or target lesion revascularization, and stroke. CHF was defined as hospitalization for worsening heart failure requiring intravenous diuretics or inotropic therapy. All clinical events were identified from electronic medical records and adjudicated by cardiologists who were not involved in the index PCI procedure. Because the number of clinical events was limited, analyses of 30-day outcomes were considered exploratory and were not designed to determine whether time-to-peak CPK independently predicts prognosis.

### Statistical analysis

Statistical analyses were performed using EZR (Easy R), a graphical user interface for R. Continuous variables were tested for normality using the Shapiro–Wilk test. Normally distributed variables were expressed as mean ± standard deviation (SD) and compared with the Student’s t-test, while non-normally distributed variables were presented as median with interquartile range (IQR) and compared using the Mann–Whitney U test. Categorical variables were expressed as numbers (percentages) and compared using the chi-square test or Fisher’s exact test. For comparisons involving more than two categories, the Kruskal–Wallis test was used.

Cumulative 30-day incidence of MACE plus CHF was evaluated using the Kaplan–Meier method, and survival curves were compared between groups using the log-rank test. A two-sided p-value < 0.05 was considered statistically significant.

## Result

### Baseline characteristics and procedural findings

A total of 239 STEMI patients were included, of whom 194 (81.2%) achieved post-PCI TIMI 3 flow (Group A) and 45 (18.8%) had TIMI 1–2 flow (Group B).

Patients in Group A had a lower prevalence of hypertension compared with Group B (70.7% vs. 87.2%, *p* = 0.037) (Table 1) and were less likely to have received angiotensin receptor neprilysin inhibitors before admission (1.5% vs. 8.9%, *p* = 0.025) (Table 2). Triglyceride levels on admission were higher in Group A than in Group B (median 147.5 mg/dL vs. 116.0 mg/dL, *p* = 0.071) (Table 1).

**Table 1:** Baseline demographic and clinical characteristics according to post-PCI TIMI flow grade

	All Patients	Group A (TIMI 3)	Group B (non-TIMI 3)	*p*-value
	(*n* = 239)	(*n* = 194)	(*n* = 45)	
Per Patient				
Age, yrs	72.0 (61.0–81.0)	72.0 (61.0–81.0)	73.0 (62.0–82.0)	0.360
Male	180 (75.3)	146 (75.3)	34 (75.6)	1.000
Body weight, kg	64.7 ± 13.2	65.0 ± 13.4	63.4 ± 12.4	0.470
BMI, kg/m^2^	24.3 (21.8–26.7)	24.6 (21.8–26.7)	23.4 (21.9–26.4)	0.435
Hypertension	177 (74.1)	138 (71.1)	39 (86.7)	0.037
Diabetes mellitus	83 (34.7)	67 (34.5)	16 (35.6)	1.000
Dyslipidemia	168 (70.3)	139 (71.6)	29 (64.4)	0.367
Smoking history	151 (63.2)	123 (63.4)	28 (62.2)	0.866
Dialysis	6 (2.5)	5 (2.6)	1 (2.2)	1.000
Myocardial Infarction	44 (18.4)	34 (17.5)	10 (22.2)	0.522
Past PCI	31 (13.0)	22 (11.3)	9 (20.0)	0.138
Past CABG	4 (1.7)	3 (1.5)	1 (2.2)	0.568
Heart failure history	20 (8.4)	14 (7.2)	6 (13.3)	0.228
CKD	111 (46.4)	85 (43.8)	26 (57.8)	0.099
PAD/AAA	6 (2.5)	3 (1.5)	3 (6.7)	0.083
Laboratory data				
NT-pro BNP, pg/mL*	199.1 (59.9–719.2)	195.5 (65.6–684.5)	227.0 (56.4–1384.3)	0.714
HbA1c, %	6.1 (5.8–6.8)	6.1 (5.8–6.8)	6.1 (5.7–6.8)	0.803
Total cholesterol, mg/dL	203.6 ± 44.0	204.7 ± 44.6	198.2 ± 41.4	0.386
LDL-C, mg/dL	126.8 ± 37.3	127.8 ± 37.7	122.8 ± 35.4	0.425
HDL-C, mg/dL	51.0 (43.0–61.0)	51.0 (43.0–61.0)	50.0 (44.8–62.8)	0.617
Triglyceride, mg/dL	141.0 (90.5–209.5)	147.5 (92.0–218.3)	116.0 (75.0–175.0)	0.071
Troponin I, pg/mL	211.7 (35.3–2545.6)	218.3 (38.3–2528.9)	211.7 (15.7–2864.6)	0.641
eGFR, mL/min/1.73 m²	62.9 (48.7–75.8)	64.5 (50.6–75.8)	53.1 (41.6–76.1)	0.177

Values are presented as mean ± SD for normally distributed variables, median (IQR) for non-normally distributed variables, and n (%) for categorical variables. NT-pro BNP values were converted from BNP using the Ishihara formula, which accounts for BMI and eGFR for log-transformed conversion between BNP and NT-pro BNP values. The non-TIMI 3 group showed a higher prevalence of hypertension and lower triglyceride levels compared with the TIMI 3 group

**Table 2:** Pre-admission medication use according to post-PCI TIMI flow grade

	All Patients	Group A (TIMI 3)	Group B (non-TIMI 3)	*p*-value
	(*n* = 239)	(*n* = 194)	(*n* = 45)	
Prehospital medications				
ACEI/ARB	77 (32.2)	63 (32.5)	14 (31.1)	1.000
ARNI	7 (2.9)	3 (1.5)	4 (8.9)	0.025
Aspirin	32 (13.4)	22 (11.3)	10 (22.2)	0.085
Diuretic	12 (5.0)	10 (5.2)	2 (4.4)	1.000
DPP-4	39 (16.3)	31(16.0)	8 (17.8)	0.823
GLP-1	4 (1.7)	2 (1.0)	2 (4.4)	0.162
Insulin	3 (1.3)	3 (1.5)	0 (0.0)	1.000
MRA	7 (2.9)	6 (3.1)	1 (2.2)	1.000
OAC	4 (1.7)	4 (2.1)	0 (0.0)	1.000
SGLT2	20 (8.4)	13 (6.7)	7 (15.6)	0.070
Statin	66 (27.6)	54 (27.8)	12 (26.7)	1.000
Thienopyridine	13 (5.4)	11 (5.7)	2 (4.4)	1.000
β-blocker	32 (13.4)	27 (13.9)	5 (11.1)	0.809

Values are presented as n (%). The use of angiotensin receptor neprilysin inhibitors (ARNI) was significantly higher in the non-TIMI 3 group compared with the TIMI 3 group, while the use of other medications was comparable between groups

*Abbreviations*: *ACEI * Angiotensin-converting enzyme inhibitor, *ARB * Angiotensin receptor blocker, *ARNI * Angiotensin receptor neprilysin inhibitor, *DPP*−4 Dipeptidyl peptidase-4 inhibitor, *GLP*−1 Glucagon-like peptide-1 receptor agonist, *MRA * Mineralocorticoid receptor antagonist, *OAC * Oral anticoagulant, *SGLT*−2 Sodium-glucose cotransporter 2 inhibitor

Procedurally, Group A had a lower rate of intra-aortic balloon pump (IABP) use (4.6% vs. 31.1%, *p* < 0.001) and distal embolization (22.7% vs. 77.8%, *p* < 0.001), with a lower trend in side branch occlusion (5.2% vs. 13.3%, *p* = 0.093). Postprocedural heart rate was significantly higher in Group A compared with Group B (80 bpm vs. 71 bpm, *p* = 0.011) (Table 3).

**Table 3:** Procedural characteristics and angiographic findings according to post-PCI TIMI flow grade

	All Patients	Group A (TIMI 3)	Group B (non-TIMI 3)	*p*-value
	(*n* = 239)	(*n* = 194)	(*n* = 45)	
PCI lesions				
Left main coronary artery	3 (1.3)	3 (1.5)	0 (0.0)	1.000
Left anterior descending artery	116 (48.5)	96 (49.5)	20 (44.4)	0.62
Left circumflex artery	43 (18.0)	37 (19.1)	6 (13.3)	0.518
Right coronary artery	86 (36.0)	66 (33.5)	21 (46.7)	0.121
Lesion length, mm	28.0 (20.0–42.0)	28.0 (20.0–41.0)	28.0 (23.0–51.0)	0.343
Operation time, min	66.5 (50.8–89.9)	63.1 (49.4–85.3)	83.9 (69.8–110.9)	< 0.001
Pre-TIMI flow grade				
0	159 (66.3)	125 (64.4)	34 (75.6)	0.113
1	31 (13.0)	25 (12.9)	6 (13.3)	
2	40 (16.7)	36 (18.6)	4 (8.9)	
3	9 (3.8)	8 (4.1)	1 (2.2)	
Final TIMI flow grade				
0	0 (0)	0 (0)	0 (0)	< 0.001
1	6(2.5)	0 (0)	6 (13.3)	
2	39 (16.3)	0 (0)	39 (86.7)	
3	194 (81.2)	194 (100)	0 (0)	
Device and technique				
IABP	23 (9.6)	9 (4.6)	14 (31.1)	< 0.001
Rotablator	4 (1.7)	4 (2.1)	0 (0.0)	1.000
IVL	1 (0.4)	1 (0.5)	0 (0.0)	1.000
DCB	25 (10.5)	18 (9.3)	7 (15.6)	0.276
Distal Protection	6 (2.5)	6 (3.1)	0 (0.0)	0.597
Procedural event				
Distal embolism	78 (32.6)	43 (22.2)	35 (77.8)	< 0.001
Side branch occlusion	17 (7.1)	11 (5.7)	6 (13.3)	0.101
Onset to visit, Door to balloon time				
< 6 h	176 (73.6)	140 (72.2)	36 (80.8)	0.503
< 12 h	42 (17.6)	37 (19.1)	5 (11.1)	
< 24 h	21 (8.8)	17 (8.7)	4 (8.9)	
Door to Balloon time, min	62.0 (50.0–81.0)	62.0 (50.0–80.0)	63.0 (49.0–102.0)	0.366
Hemodynamic State				
Preoperative systolic blood pressure, mmHg	158.0 (133.5–174.5)	159.0 (135.0–176.5)	153.0 (128.0–166.0)	0.220
Preoperative diastolic blood pressure, mmHg	91.7 ± 20.1	92.1 ± 20.3	89.6 ± 19.6	0.456
Preoperative heart rate, bpm	75.0 (63.0–91.0)	75.0 (65.0–92.0)	73.0 (62.0–83.0)	0.443
Postoperative systolic blood pressure, mmHg	137.6 ± 25.3	138.6 ± 26.1	133.0 ± 21.2	0.182
Postoperative diastolic blood pressure, mmHg	81.4 ± 14.5	81.9 ± 14.5	79.3 ± 14.8	0.283
Postoperative heart rate, bpm	80.0 (69.0–91.0)	80.5 (71.0–91.0)	71.0 (64.0–88.0)	0.010

Values are presented as mean ± SD, median (IQR), or n (%). Group A: post-PCI TIMI 3 flow; Group B: post-PCI non-TIMI 3 flow. Patients in Group B had significantly higher rates of intra-aortic balloon pump (IABP) use and distal embolism compared with Group A, while other procedural and angiographic variables were generally comparable between groups

*Abbreviations*: *IABP * Intra-aortic balloon pump, *Rota * Rotational atherectomy, *IVL * Intravascular lithotripsy, *DCB * Drug-coated balloon

### Time to peak CPK and peak CPK levels

**Fig. 2:**
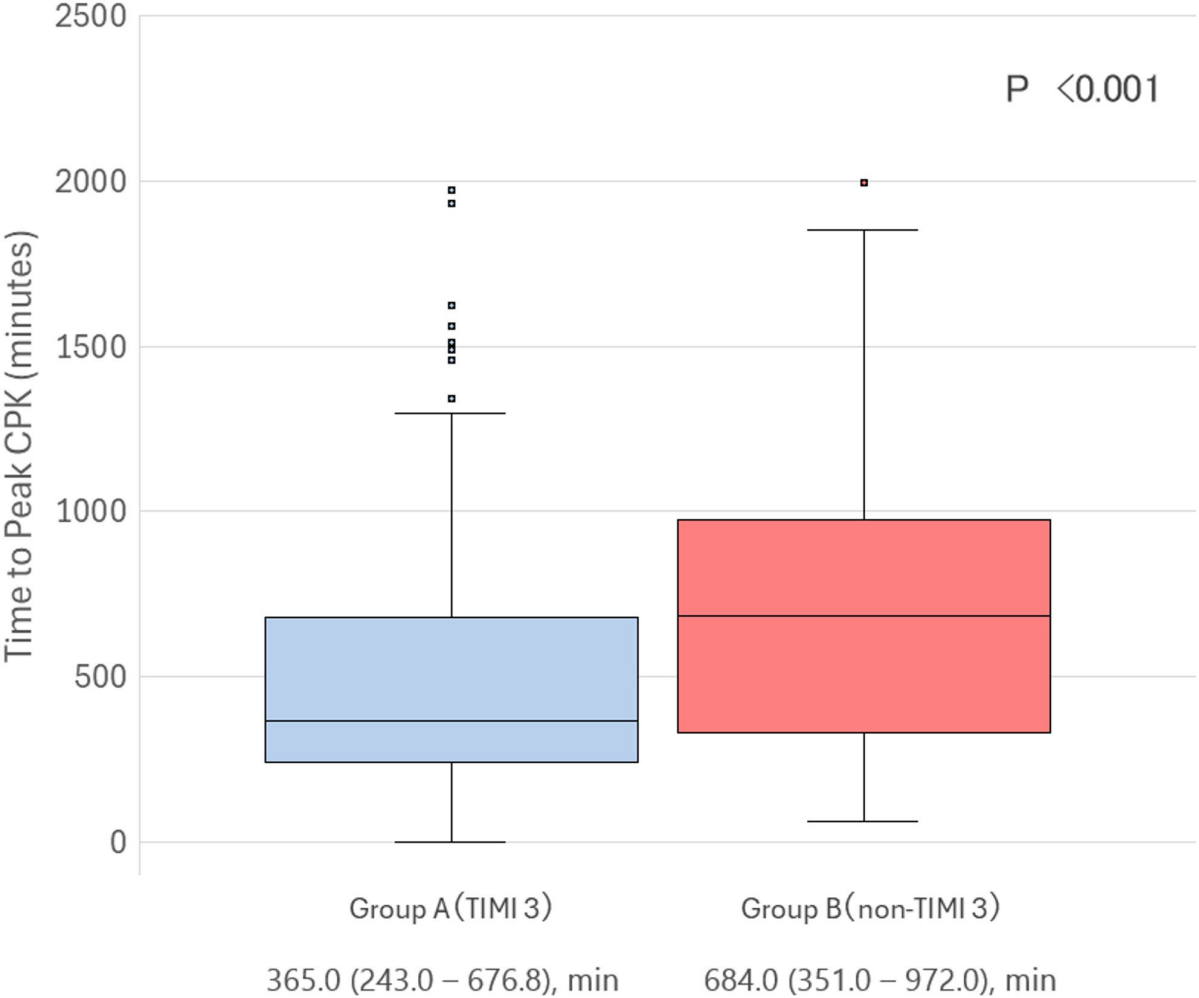
Time to peak CPK according to post-PCI TIMI flow. Box-and-whisker plot showing the distribution of time to peak creatine phosphokinase (CPK) in patients with post-PCI TIMI 3 flow and non-TIMI 3 flow. Median time to peak CPK was significantly shorter in the TIMI 3 group compared with the non-TIMI 3 group (365.0 [243.0–676.8] min vs. 684.0 [351.0–972.0] min, *p* < 0.001, Mann–Whitney U test)

The median time to peak CPK was significantly shorter in Group A compared with Group B (365.0 [243.0–676.8] min vs. 684.0 [351.0–972.0] min, *p* < 0.001) (Table 4; Fig. 2).

**Table 4:** Comparison of post-PCI creatine phosphokinase (CPK) kinetics according to final TIMI flow grade

	All Patients	Group A (TIMI 3)	Group B (non-TIMI 3)	*p*-value
	(*n* = 239)	(*n* = 194)	(*n* = 45)	
Time to peak CPK, min	408.0 (251.5–759.5)	365.0 (243.0–676.8)	684.0 (351.0–972.0)	< 0.001
Peak CPK, IU/L	2282.0 (1233.5–3921.5)	2164.0 (1148.8–3662.8)	2542.0 (1665.0–5303.0)	0.058

Values are presented as median (IQR). Group A: post-PCI TIMI 3 flow; Group B: post-PCI non-TIMI 3 flow. The time to peak CPK was significantly longer in Group B compared with Group A, while peak CPK levels did not differ significantly between the groups

*Abbreviations*: *CPK * Creatine phosphokinase, *TIMI * Thrombolysis in Myocardial Infarction, *PCI * Percutaneous coronary intervention, *IQR * Interquartile range

In contrast, peak CPK levels were not significantly different between Group A and Group B (2164.0 [1148.8–3662.8] IU/L vs. 2542.0 [1665.0–5303.0] IU/L, *p* = 0.058).

To complement these findings, CK-MB kinetics were assessed in 232 patients with available measurements (Supplementary Table S1). Similar to total CPK, the time to peak CK-MB was significantly shorter in Group A than in Group B (325.0 [213.0–528.0] min vs. 519.0 [261.0–799.5] min, *p* = 0.002), whereas peak CK-MB levels did not differ significantly between the groups. These consistent results reinforce the association between impaired reperfusion and delayed enzymatic washout.

### Thirty-day outcomes

**Fig. 3:**
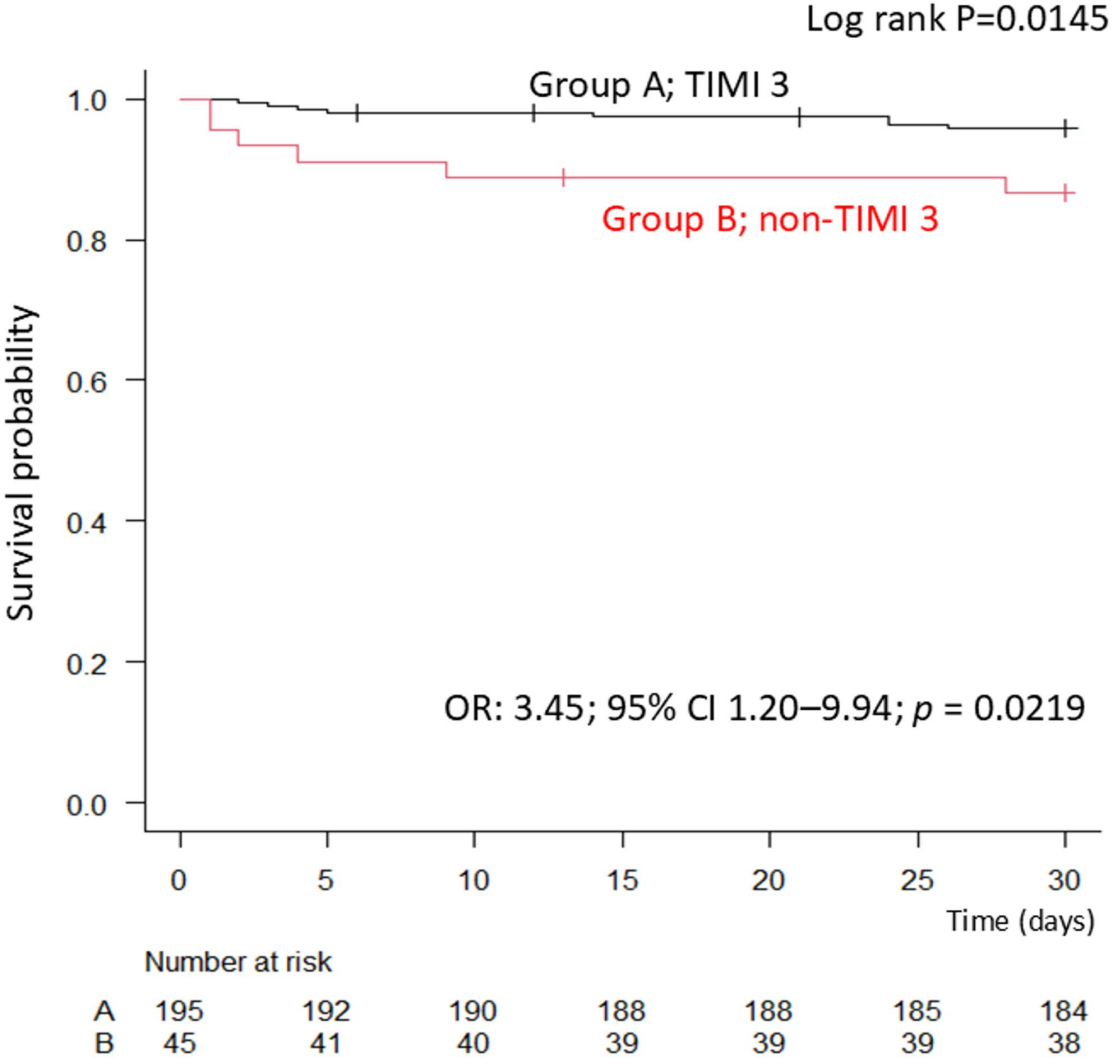
Kaplan–Meier curves for 30-day cumulative incidence of MACE plus congestive heart failure Kaplan–Meier curves showing the 30-day cumulative incidence of major adverse cardiovascular events (MACE) plus congestive heart failure (CHF), stratified by final post-PCI TIMI flow grade (TIMI 3 vs. non-TIMI 3). Event-free survival was significantly lower in the non-TIMI 3 group compared with the TIMI 3 group (log-rank *p* = 0.01)

At 30 days, the cumulative incidence of MACE plus CHF was significantly lower in Group A compared with Group B (4.1%, 8/194 vs. 13.3%, 6/45) (Table 5). Kaplan–Meier survival analysis showed a significantly higher event-free survival rate in Group A (log-rank *p* = 0.01) (Fig. 3).

**Table 5:** Thirty-day clinical outcomes according to post-PCI TIMI flow grade

	Group A (TIMI 3)	Group B (non-TIMI 3)	*p*-value
	(*n* = 194)	(*n* = 45)	
30-day MACE + CHF events, %	8 (4.1)	6 (13.3)	0.01 (Log-rank)

Values are presented as n (%). Group A: post-PCI TIMI 3 flow; Group B: post-PCI non-TIMI 3 flow. The cumulative incidence of major adverse cardiovascular events (MACE) plus congestive heart failure (CHF) at 30 days was significantly higher in Group B compared with Group A

*Abbreviations*: *TIMI * Thrombolysis in Myocardial Infarction, *PCI * Percutaneous coronary intervention, *MACE * Major adverse cardiovascular events, *CHF * Congestive heart failure

## Discussion

Achieving TIMI 3 flow has been established as the primary goal of primary PCI in STEMI patients, as it is strongly associated with improved myocardial salvage and clinical outcomes [9–11]. In the present study, we demonstrated that STEMI patients with suboptimal post-PCI flow (TIMI 1–2) experienced a significant delay in the time to CPK peak compared with those who achieved TIMI 3 flow. This finding suggests that CPK peak timing reflects differences in reperfusion quality in the contemporary PCI era.

While TIMI 3 flow is widely accepted as an angiographic endpoint, it does not always reflect microvascular integrity. No-reflow and distal embolization can occur even in patients with angiographic TIMI 3 flow, and these phenomena are strongly linked to adverse outcomes [12]. The timing of CPK peak may therefore provide complementary information regarding microvascular reperfusion at the tissue level. In our study, distal embolism was markedly more frequent in the non-TIMI 3 group, supporting its role as a major mechanism of impaired washout and delayed CPK peak. The higher rate of IABP use in this group further indicates greater hemodynamic compromise, consistent with more extensive myocardial damage. Moreover, longer procedural times and lower postoperative heart rates in the non-TIMI 3 group may reflect procedural complexity and impaired myocardial recovery, although their direct effects on biomarker kinetics remain less certain. Our observation that peak CPK values were not significantly different between groups supports the notion that the timing, rather than the absolute magnitude, of enzymatic release may better capture the adequacy of reperfusion. These results support the concept that enzymatic biomarker kinetics are determined not only by infarct size but also by the quality of myocardial reperfusion [2]. A supplementary analysis also demonstrated delayed CK-MB peak timing in the non-TIMI 3 group, indicating that impaired enzymatic washout is consistent across biomarkers. Because CK-MB sampling was not performed at fixed intervals, its timing could not be assessed with the same precision as total CPK and was therefore used only as a complementary measure.

Although peak timing provides insight into reperfusion dynamics, the enzymatic area under the curve (AUC) may more directly reflect total myocardial necrosis. However, because later CPK sampling intervals varied among patients, reliable AUC calculation was not feasible in this retrospective dataset. Future studies with fully standardized sampling schedules will be necessary to determine whether AUC offers incremental value beyond peak timing alone.

Importantly, our study also showed that patients with non-TIMI 3 flow had a significantly higher incidence of 30-day MACE plus CHF. However, this outcome analysis was exploratory and limited by the small number of events; therefore, the prognostic implications of CPK kinetics should be interpreted with caution. Because the study was not powered to evaluate whether time-to-peak CPK is independently associated with clinical outcomes, our findings do not establish CPK kinetics as a prognostic marker. Rather than serving as a standalone risk-stratification tool, time-to-peak CPK may function as an adjunctive indicator of tissue-level reperfusion that complements, but does not replace, angiographic assessment in routine practice.

Previous studies in the PCI era have also examined enzymatic biomarker kinetics. Shibahashi et al. demonstrated that delayed balloon-to-peak CK-MB time was prognostically relevant even after successful angiographic reperfusion [13], whereas Katayama et al. associated delayed CK-MB peak with impaired left ventricular recovery in patients achieving TIMI 3 flow [14]. Elakabawi et al. identified predictors of suboptimal angiographic reperfusion but did not address biomarker kinetics [15]. In contrast, our study used total CPK rather than CK-MB, defined peak timing from the end of PCI, directly compared TIMI 3 versus non-TIMI 3 flow, and assessed 30-day outcomes. Taken together, these findings extend prior work by showing that delayed CPK peak is closely linked to suboptimal reperfusion and may provide supportive information alongside angiographic assessment in contemporary STEMI care.

### Limitation

This study has several limitations.

First, it was a retrospective, single-center study with a limited sample size, which may restrict the generalizability of our findings.

Second, although CPK levels were measured systematically with frequent early sampling, variability in sampling intervals might have affected the precise determination of peak values. Furthermore, the sampling frequency reflects institutional practice and may differ across centers, which may limit the generalizability of the biomarker kinetic findings.

Third, both time-to-peak CPK and post-PCI TIMI flow were used as surrogate markers of reperfusion quality; however, neither fully reflects microvascular integrity. Because myocardial blush grade and other advanced imaging modalities were not routinely documented, direct assessment of microvascular perfusion was not feasible.

Fourth, although CK-MB was available in nearly the entire cohort and showed results consistent with total CPK, it was not obtained at identical time intervals to CPK and therefore could not be included as a primary kinetic marker.

Fifth, clinical outcomes were assessed only up to 30 days, and the longer-term prognostic significance of delayed CPK peak remains to be determined.

Sixth, the number of 30-day clinical events was relatively small, which limited the statistical power for outcome analysis and precluded reliable multivariable adjustment. Therefore, the findings related to MACE plus CHF should be interpreted as exploratory and hypothesis-generating.

Seventh, although CPK was sampled frequently in the early phase after PCI, later measurements were not obtained at uniform intervals across all patients. This variability prevented reliable calculation of the enzymatic AUC, which may better reflect total myocardial necrosis. Therefore, the potential incremental value of AUC analysis could not be assessed in this study.

## Conclusion

In STEMI patients undergoing primary PCI, suboptimal post-PCI TIMI flow was associated with delayed time to peak CPK and higher incidence of 30-day adverse events. The observed delay in CPK peak may reflect impaired myocardial reperfusion; however, its clinical utility as an independent marker remains exploratory. Further prospective studies are needed to validate whether CPK kinetics can provide additional insight beyond angiographic assessment in contemporary STEMI care.

## Data Availability

The datasets generated and/or analyzed during the current study are not publicly available due to patient privacy and institutional regulations but are available from the corresponding author on reasonable request.

## Abbreviations

BMI: Body mass index
PCI: Percutaneous coronary intervention
CABG: Coronary artery bypass grafting
CKD: Chronic kidney disease
PAD: Peripheral artery disease
AAA: Abdominal aorta aneurysm
BNP: Brain natriuretic peptide
HbA1c: Glycated hemoglobin
LDL-C: Low-density lipoprotein cholesterol
HDL-C: High-density lipoprotein cholesterol
eGFR: Estimated glomerular filtration rate
